# Metabolic Assessment in Human Pluripotent Stem Cell‐Derived Cerebral Organoids Using HR‐MAS NMR Spectroscopy

**DOI:** 10.1002/nbm.70343

**Published:** 2026-06-26

**Authors:** Maria Alejandra Castilla Bolanos, Vorapin Chinchalongporn, Rajshree Ghosh Biswas, Colleen Bailey, Maggie Wu, Ronald Soong, Fermisk Saleh, Andre Simpson, Carol Schuurmans, Jamie Near

**Affiliations:** ^1^ Sunnybrook Research Institute Toronto Ontario Canada; ^2^ Department of Medical Biophysics University of Toronto Toronto Ontario Canada; ^3^ Department of Chemistry University of Toronto Toronto Ontario Canada; ^4^ Department of Biochemistry University of Toronto Toronto Ontario Canada

**Keywords:** cerebral organoids, human brain model, human pluripotent stem cells, metabolism, NMR spectroscopy

## Abstract

Brain metabolism is vital to healthy brain function and is often altered in disease; yet direct investigation in patients is challenging. Although animal models are commonly used for studying brain metabolism, their use is under increasing scrutiny due to concerns of animal welfare and model validity. Human pluripotent stem cell (hPSC)‐derived cerebral organoids (COs) present a unique opportunity to model human brain developmental and neuropathological processes, allowing for detailed metabolic characterization via multiple approaches. Here, we applied high‐resolution magic‐angle spinning (HR‐MAS) proton nuclear magnetic resonance (^1^H‐NMR) spectroscopy to analyze metabolite levels in hPSC‐derived COs, establishing a pipeline to study neurometabolic pathways in these engineered human brain tissues. We identified and quantified 17 metabolites in hPSC‐derived COs at different stages of maturity. The high spectral quality (linewidth < 4 Hz, SNR > 65) allowed detection of metabolite levels in 85‐ to 312‐day‐old hPSC‐derived COs, which exhibited a metabolic profile similar to human fetal brain, with key distinguishing features relative to human adult brain, including: elevated lactate levels; approximately equimolar glutamate and glutamine levels; low *N*‐acetylaspartate levels; and an abundance of hypotaurine. In summary, this study presents direct metabolic assessment in intact COs via HR‐MAS ^1^H‐NMR spectroscopy. Our approach provides a platform for investigating human brain metabolism and its alteration in human brain models of neurodegeneration.

## Graphical abstract

We present a direct method for evaluating metabolism in intact hPSC‐derived cerebral organoids (COs) via HR‐MAS ^1^H‐NMR spectroscopy. This approach suggests that COs exhibit a metabolic profile similar to *in vivo* human fetal brain, with high levels of lactate and hTau, low levels of NAA, and equimolar levels of Glu and Gln. This study proposes HR‐MAS NMR spectroscopy as a useful methodology for assessing neurochemistry and metabolism in human brain models. This approach will enable studies to understand how brain metabolism is impacted by specific genetic and gene transcriptional alterations.

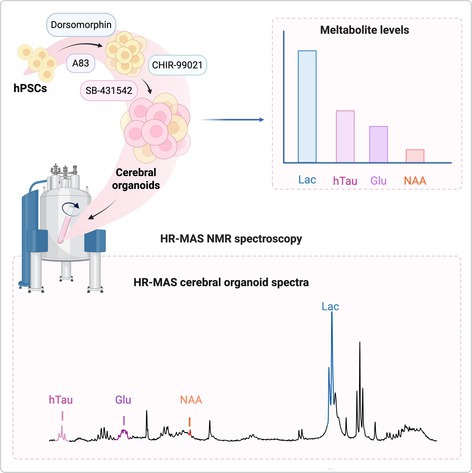

**Abbreviations**
Ace: acetate
Ala: alanine
Cho: choline
Cr: creatine
CO: cerebral organoid
etOH: ethanol
GABA: gamma‐aminobutyric acid
Glc: glucose
Gln: glutamine
Glu: glutamate
Gly: glycine
GPC: glycerophosphocholine
GSH: glutathione
HR MAS NMR: high‐resolution magic angle spinning nuclear magnetic resonance
hTau: hypotaurine
Lac: lactate
Leu: leucine
Lys: lysine
mIns: myo‐inositol
NAA: *N*‐acetylaspartate
PCh: phosphocholine
PCr: phosphocreatine
Pt: putrescine
sIns: scyllo‐inositol
Tau: taurine
Val: valine

## 1 Introduction

With animal research under increasing scrutiny in many jurisdictions due to concerns related to animal welfare and scientific validity [1, 2], alternative model systems are needed. Human pluripotent stem cell (hPSC)‐derived cerebral organoids (COs) represent a breakthrough in neuroscience, allowing human brain tissue—normally difficult for researchers to access—to be examined in a laboratory [3]. COs overcome some limitations of the commonly studied rodent brain, which fundamentally differs from the human brain, both genetically and structurally [4]. Moreover, COs have been used to effectively model human diseases such as Alzheimer's disease and other types of dementia [5].

Investigating metabolism is essential for understanding brain health, as metabolic and neurochemical abnormalities underlie neurodegenerative diseases such as amyotrophic lateral sclerosis and Alzheimer's disease [6, 7]. Although metabolic alterations in disease can be inferred indirectly via transcriptomic analyses [8], *in situ* nuclear magnetic resonance (NMR) spectroscopy may provide a more direct approach [9]. Proton (^1^H) NMR also offers a key translational advantage in that it can readily be performed in human patients via *in vivo* magnetic resonance spectroscopy (MRS). However, to our knowledge, ^1^H‐NMR measurements have yet to be acquired in human‐derived COs. Here, we report the use of high‐resolution magic‐angle spinning (HR‐MAS) NMR spectroscopy [10] for metabolic analysis of intact hPSC‐derived COs at different stages of maturity. Using this approach, we detected and quantified 17 metabolites relevant to the human brain. The hPSC‐derived CO neurochemical profile showed some similarities to that of *in vivo* fetal human brain, in agreement with previous studies, suggesting that COs serve as a model of early fetal brain development [11].

## 2 Materials and Methods

### 2.1 HR‐MAS NMR Spectroscopy of COs

**FIGURE 1:**
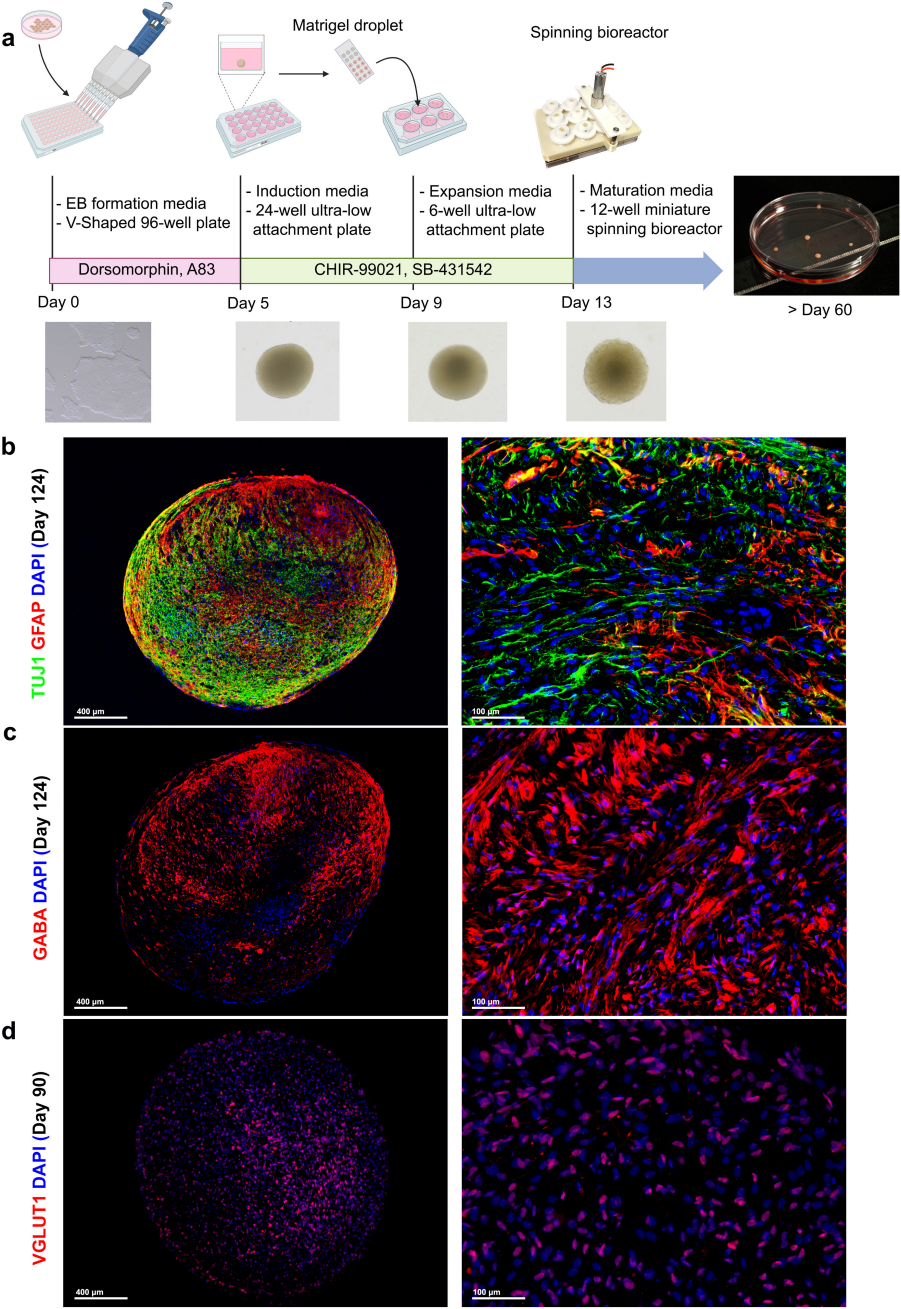
Generation and neuronal identity of hPSC–derived COs. (A) Schematic overview of the generation of COs from hPSCs using the STEMdiff Cerebral Organoid Kit. (B, C) 124‐day‐old CO co‐immunolabeled for TUJ1 (neuronal marker; green) and GFAP (astrocytic marker, red), and GABA neurotransmitter (a marker of GABAergic/inhibitory neurons, red). (D) 90‐day‐old CO immunolabeled for vesicular glutamate transporter VGLUT1 (a marker of glutamatergic/excitatory neurons, red). Blue is the DAPI nuclear counterstain in all images. Scale bars are 400 μm (left) and 100 μm (right).

Twelve hPSC‐derived COs were generated using a modified dual Smad inhibition protocol [12], and their neural identity was verified by immunohistochemistry (Figure 1). CO generation and immunostaining protocols are provided in the Supporting Information. Each CO was scanned at a different maturity stage ranging from 85 to 312 days (one replicate per age) using HR‐MAS NMR spectroscopy, with protocols adapted from Fortier‐McGill [13]. All NMR experiments were performed on a Bruker Avance III 500 MHz ^1^H spectrometer (Bruker, Billerica MA, USA), equipped with a 4 mm ^1^H‐^13^C‐^15^N (^2^H lock) comprehensive multiphase (CMP) NMR probe (Bruker Switzerland AG, Fällanden, Switzerland) fitted with a magic angle gradient. For each scan, a single organoid was positioned in a 4 mm reduced volume (50 μL) rotor (HZ07213, Bruker Billerica, MA, USA) to maintain it within in the sensitive region of the probe. All experiments were performed at 5°C and locked on D_2_O. During the experiment, perfusion of oxygen and cell culture media was not maintained. Samples were spun at the magic angle at a relatively slow spin rate of 2500 Hz to prevent the rupture of biological tissue [14, 15] while still positioning the spinning sidebands of water outside the spectral window. ^1^H experiments spectra were collected using 16,384 time‐domain points, a spectral width of 20 ppm, 8 dummy scans and 512 total scans. Presaturation using an effective B_1_ field of 100 Hz was prepended to all experiments. Experiments used for quantitation were performed with a repetition time of 5× T1 of the sample (~11.82 s) between subsequent scans. Total scan duration for each organoid was approximately 220 min. Further experimental details are shown in Table S1, according to the recommended reporting standards for *in vivo* MRS [16]. Due to the low number of replicates at each organoid maturity stage, we were not able to reliably assess metabolic changes as a function of organoid maturity (see Section 5).

### 2.2 NMR Spectral Editing

**FIGURE 2:**
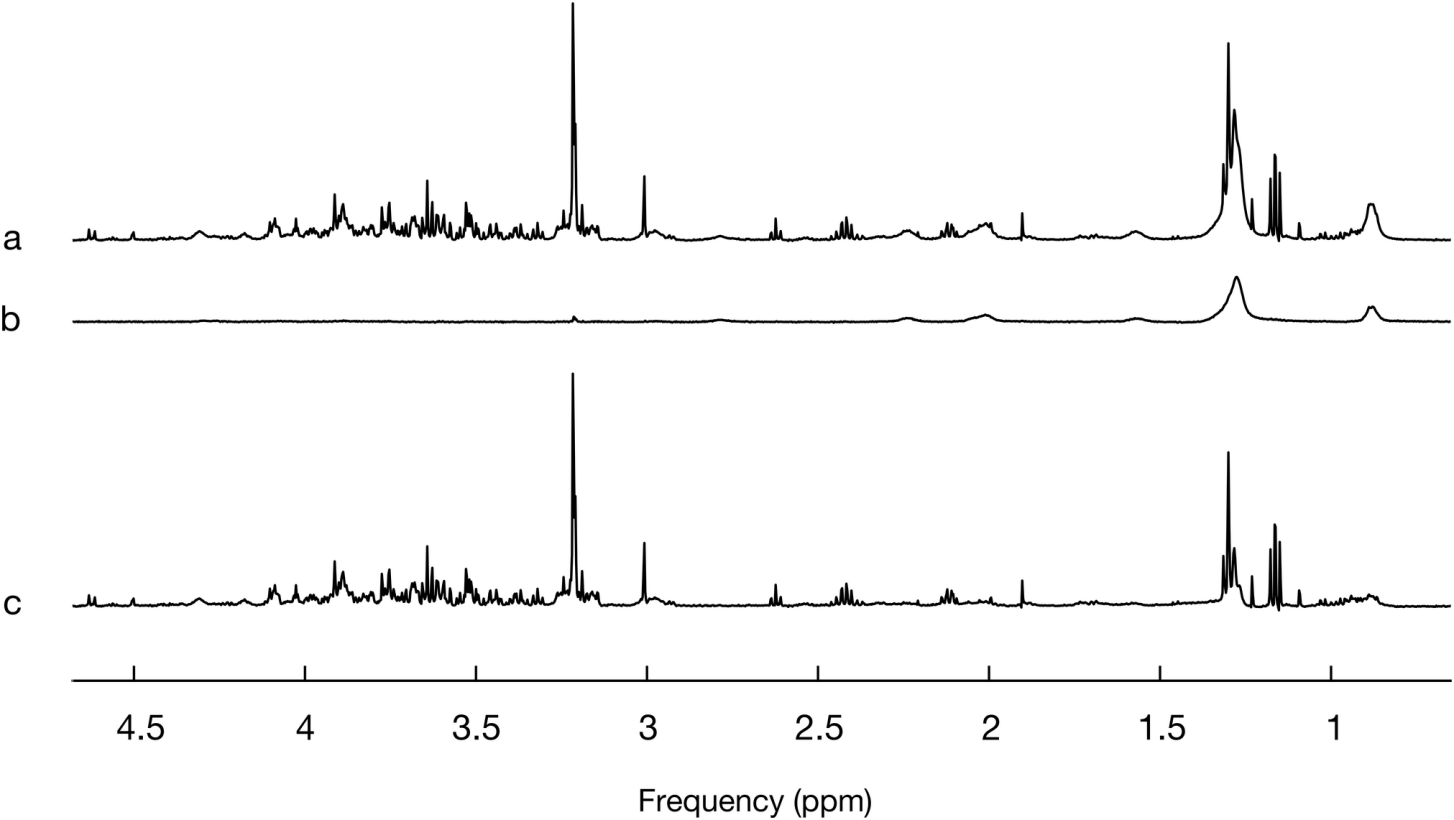
Diffusion edited HR‐MAS spectra. hPSC‐derived cerebral organoids HR‐MAS spectra (A) were subjected to diffusion editing for removal of macromolecular resonances (B). The resulting inverse diffusion‐edited spectra consist of only freely diffusing metabolites (C).

Prominent and broad resonances from macromolecules in biological tissues can interfere with quantification of the narrower metabolite resonances of interest. To isolate the mobile metabolite fraction, an inverse diffusion editing (IDE) approach was employed as previously described [17, 18]. Briefly, a strongly diffusion‐weighted spectrum (DE: diffusion gradient on, depicting only macromolecular components/components with restricted diffusion) was scaled and subtracted from a non‐diffusion weighted spectrum (diffusion gradient off, depicting both components with restricted and non‐restricted diffusion, e.g., both macromolecules and metabolites). Diffusion editing experiments were acquired using a bipolar pulse pair with longitudinal eddy‐current delay (BPP‐LED) diffusion sequence [19]. Diffusion weighted acquisitions used a 2.4 ms encode/decode gradient pulse with an amplitude of 60 G/cm and a diffusion delay of 120 ms. Scaling and subtraction were performed in MATLAB 2023b using the FID‐A toolkit [20]. To minimize noise amplification due to the subtraction process, we replaced the trailing noise at the end of the diffusion‐weighted FID with zeros prior to scaling and subtraction. The resulting diffusion edited spectrum depicts only the small molecule signals from mobile metabolites (Figure 2).

### 2.3 HR‐MAS NMR Metabolite Assignment and Quantification

Metabolites were assigned using the Human Metabolome Database (hmdb.ca), and by alignment with simulated metabolite model spectra generated in FID‐A using literature values for chemical shifts and J‐coupling [20]. Peak integration was performed using TopSpin 4.3 (Bruker), and absolute quantification was calculated by referencing to an external standard consisting of 50 μL of a 10 mM alanine solution, acquired using the identical protocol. Quantitative metabolite concentrations, corrected for organoid volume as well as the number of protons contributing to the integrated NMR peak, were estimated according to the following formula:

(1) $$\left[C_{i}\right]=C_{\mathrm{ref}}\cdot \frac{\;I_{i}\;}{I_{\mathrm{ref}}\;}\cdot \frac{\;{\mathrm{NP}}_{\mathrm{ref}}\;}{\;{\mathrm{NP}}_{i}\;}\cdot \frac{\;V_{\mathrm{ref}}\;}{\;V_{i}\;}$$

where $C_{i}$ refers to the metabolite concentration; $C_{\mathrm{ref}}$ refers to the alanine standard concentration; $I_{i}$ is the integral of the metabolite i within the pre‐defined integration region; $I_{\mathrm{ref}}$ is the integral of the methyl doublet of the alanine standard spectrum; ${\mathrm{NP}}_{\mathrm{ref}}$ is the number of protons contributing to the alanine standard methyl doublet signal (${\mathrm{NP}}_{\mathrm{ref}}=3$); ${\mathrm{NP}}_{i}$ is the number of protons contributing to the integrated metabolite signal; and $V_{\mathrm{ref}}$ is the volume of the alanine standard ($V_{\mathrm{ref}}=50\;\mathrm{\mu L}$). The organoid volume, $V_{i}$, was estimated by measuring the organoid mass using an analytical balance and converting the mass to volume using an assumed density of 1 g/mL. Table S2 displays the estimated metabolite concentration in 12 hPSC‐derived COs.

## 3 Results

### 3.1 Generation of COs

At Days 18 and 30, hPSC‐derived COs had neural rosette‐like structures, with an inner ventricular zone labeled by SOX2, a neural progenitor cell marker. TUJ1+ immature neurons were dispersed throughout the CO rosettes, but progressively migrated outwards, forming an outer layer above the ventricular‐like zones of each rosette (Figure S1). By Day 124, rosettes had largely resolved, and neurons (identified by TUJ1 and DAPI) and astrocytes (identified by GFAP) intermingled throughout the CO, with overlapping processes (Figure 1B), consistent with brain‐like interactive networks. To assess neuronal phenotypes, we observed GABA‐ergic interneurons (Figure 1C) and VGLUT1/SLC17A7‐expressing glutamatergic pyramidal neurons (Figure 1D), confirming the presence of both inhibitory and excitatory neuronal types. Thus, mature COs are composed of a heterogeneous mix of at least three neural cell types: astrocytes, excitatory neurons, and inhibitory neurons.

### 3.2 HR‐MAS NMR Spectroscopy in COs

**FIGURE 3:**
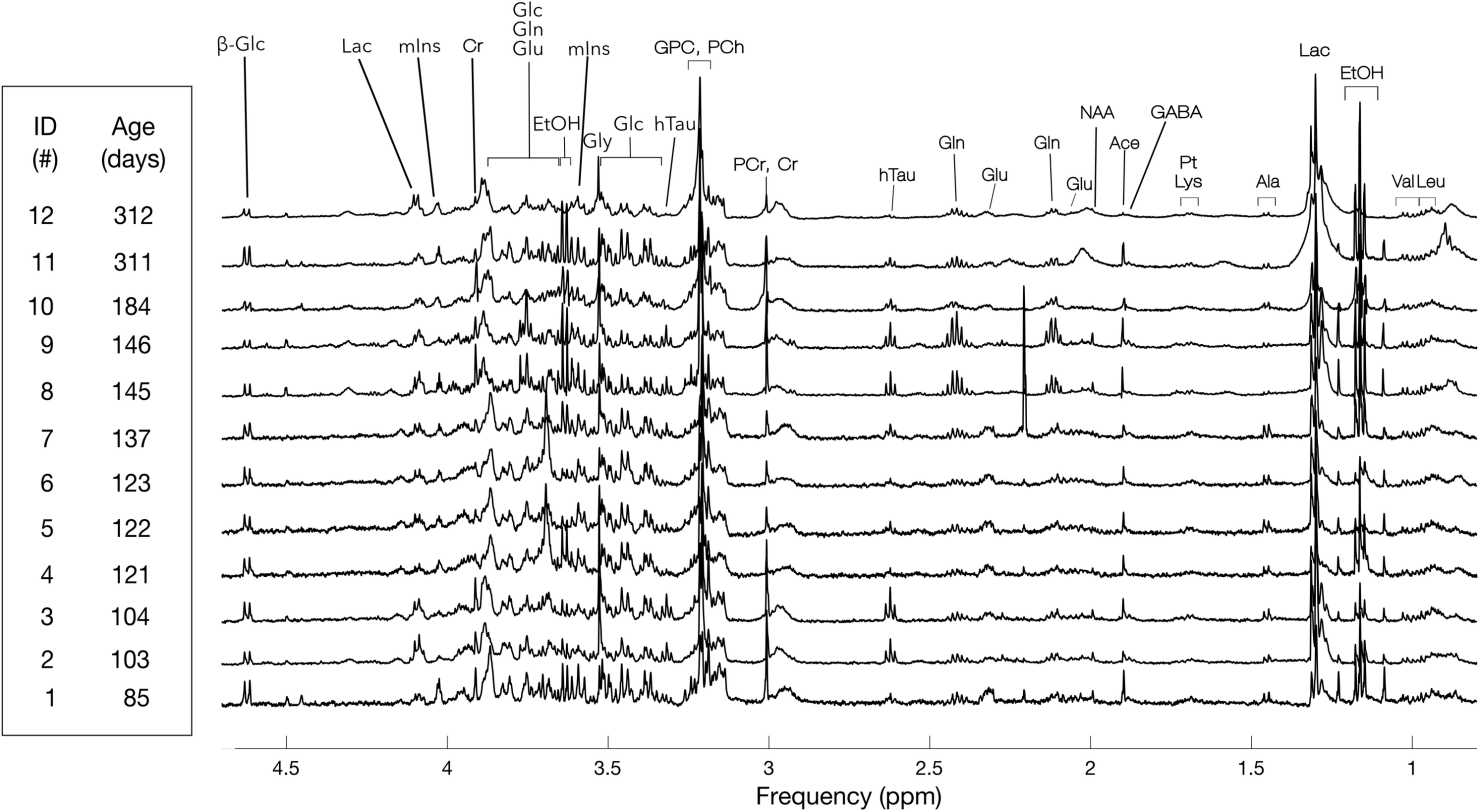
^1^H HR‐MAS NMR spectra of 12 intact hPSC‐derived COs. Metabolite peak assignments are indicated above the spectra. For each organoid, 512 scans were acquired and averaged to produce a single, high‐fidelity spectrum (*n* = 1 organoid per age). CO ID and corresponding ages are listed in table on the left. Spectra were aligned and scaled to total spectral power over the spectral range shown. Ace: acetate; Ala: alanine; Cr: creatine; etOH: ethanol; GABA: γ‐hydroxybutyric acid; Gln: glutamine; Glu: glutamate; Gly: glycine; GPC: glycerophosphocholine; hTau: hypotaurine; Lac: lactate; Leu: leucine; Lys: lysine; mIns: myo‐inositol; NAA: *N*‐acetylaspartate; PCh: phosphocholine; Pt: putrescine; Val: valine; β‐Glc: glucose.

**FIGURE 4:**
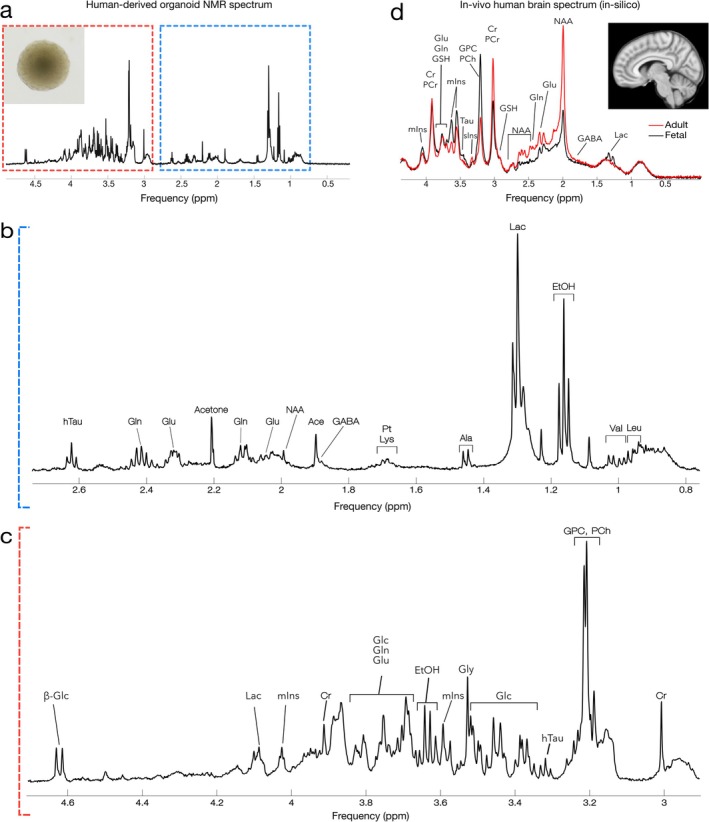
^1^H HR‐MAS NMR spectroscopy in hPSC–derived COs. (A) Average spectrum of 12 hPSC–derived COs from 85 to 312 days old scanned using a Bruker Avance III 500 MHz ^1^H spectrometer with a comprehensive multiphase NMR probe fitted with a magic‐angle gradient at 278 K and locked on D_2_O. (B, C) Zoomed region between 0.8–2.6 and 3.0–4.6 ppm of average spectrum of 12 hPSC–derived COs from 85 to 312 days of maturation. (D) Synthetic *in vivo* adult (red) and fetal (black) human brain spectra generated in silico illustrate the most prominent metabolites in human brain tissue, including NAA, Cr, GPC, PCh, and Glu.

High‐quality NMR spectra with narrow metabolite linewidths (max linewidth = 3.7 Hz, mean = 2.7 ± 0.6 Hz) and high signal‐to‐noise ratios (min SNR = 67, mean = 193 ± 153) were obtained. Spectral quality and appearance are remarkably reproducible from all 12 hPSC‐derived COs aged 85–312 days (Figure 3). A spectrum averaged across all organoids is shown in Figure 4 and shows 20 assigned metabolites in the upfield region (0.8–4.7 ppm, Figure 4C). Two synthetic *in vivo* brain spectra were generated in FID‐A for comparison, one representing adult human brain (Figure 4D, red curve), and one representing fetal human brain (Figure 4D, black curve). Individual metabolite ^1^H NMR basis spectra were simulated in FID‐A [20], and scaled according to typical metabolite concentrations observed in adult human brain (based on Govindaraju et al. [21] and de Graaf [22]), and fetal human brain (based on Tomiyasu et al. [23], Hüppi et al. [24], and Kreis et al. [25]). Scaled metabolite basis functions were then combined to produce representative spectra for comparison with our experimental data. Many of the most abundant metabolites in the human brain were present in COs. We identified the following metabolites (in order of decreasing concentration): lactate, myo‐inositol, ethanol, glutamate (Glu), glutamine (Gln), glycine, acetate, putrescine, phosphocholine, glycerophosphocholine, choline, hypotaurine (hTau), creatine, GABA, *N*‐acetylaspartate (NAA), alanine, valine, leucine, and glucose (alpha and beta).

### 3.3 Neurometabolite Levels

Figure S2 shows the estimated metabolic concentrations per organoid. Table S3 presents the mean CO metabolite concentrations in comparison with literature values from fetal and adult human brain. Adult reference concentrations were calculated from ranges reported by Govindaraju et al. [21] and de Graaf et al. [22], whereas fetal measurements represent averages derived from Tomiysasu et al. [23], Hüppi et al. [24] and Kreis et al. [26].

COs showed consistently high lactate levels, contrasting with the typical low lactate levels of healthy adult human brain (Figure 4). All 12 COs showed strong Gln resonances at 2.41 and 2.11 ppm, whereas Glu appeared as a less distinct multiplet at 2.32 ppm (Figure 3). Whereas a high Glu:Gln ratio of approximately 5:1 is typically observed in adult human and rat brain, COs exhibited approximately equimolar levels of Glu and Gln, as shown in Figures 3 and 4.

One of the most abundant metabolites in adult human and rodent brain *in vivo* is NAA, which appears as a prominent singlet at 2.0 ppm (Figure 4D). In sharp contrast to adult human brain *in vivo*, NAA was detected at very low levels in COs (Figure 4B). Moreover, the putative NAA peak appeared slightly below 2.0 ppm, indicating a lower chemical shift in the *in vitro* environment, or possibly the presence of another *N*‐acetyl component. NAA acts as a neuronal osmolyte, providing acetate for myelin synthesis in oligodendrocytes, and supporting mitochondrial metabolism [27]. The low NAA levels in COs resemble an early developmental stage in the human fetal brain, wherein NAA levels remain low until 22–24 gestational weeks [28].

We observed a pair of strong triplet resonances at 2.62 and 3.34 ppm, which were assigned to hTau (Figure 4A–C). This finding was of interest because hTau is typically absent in *in vivo* adult human brain spectra (although it has been observed previously in cultured oligodendrocytes [21]). In contrast, taurine, a related metabolite that is abundant in adult human and rodent brain, was undetected in all COs.

## 4 Discussion

To our knowledge, this is the first study investigating the metabolism of human hPSC‐derived COs via ^1^H HR‐MAS NMR spectroscopy. HR‐MAS is a routine tool in solid‐state NMR and has been used to assess metabolite levels in intact brain and other tissues [10]. We used a spinning rate of 2500 Hz, which sufficiently removes line broadening and avoids spinning sidebands while preserving tissue integrity [14]. Our findings suggest that hPSC‐derived COs exhibit a metabolic profile similar to the human brain *in vivo*, with distinguishing characteristics that include (1) elevated lactate, (2) approximately equimolar Glu and Gln levels, (3) low NAA, and (4) the presence of hTau (and absence of taurine). Some of these features, namely, elevated lactate [23, 25], decreased NAA [23, 24, 25], and low taurine [24, 25], suggest an early developmental phenotype similar to the human fetal brain.

The finding of high lactate suggests enhanced glycolysis, which is typical of rapidly growing tissues, though potentially exacerbated by limited oxygen availability at the CO core and during NMR acquisition. Prominent phosphocholine and glycerophosphocholine resonances were also observed in hPSC‐derived CO NMR spectra, which are also characteristic of growing tissues with restricted oxygen availability [28].

We observed approximately equimolar Glu and Gln levels in COs compared with the 5:1 Glu:Gln ratio typically observed in adult brain *in vivo*. In the mammalian central nervous system, Glu is the primary excitatory neurotransmitter, whereas Gln is synthesized in glial cells. The observed equimolar levels may result from immature astrocytes and underdeveloped astrocyte‐neuron interactions in COs [29]. Other factors, such as reaction kinetics, enzyme activity levels, and nutrient diffusion may influence Glu/Gln interaction, resulting in a low Glu concentration. Future research should explore these interactions to understand how this balance is maintained.

Low NAA levels in hPSC‐derived COs contrasts with the high levels observed in adult human and rodent brain, but aligns with findings in fetal brain [28], supporting the use of COs as models for early brain development. NAA metabolism involves complex interactions between neural cell types. Neurons produce NAA, but its synthesis depends on aspartate from astrocytes. NAA is hydrolyzed by ASPA in oligodendrocytes, which emerge slowly in COs after three months [30]. Oligodendrocytes were not identified before 124 days post‐generation, explaining NAA absence in earlier stages. This lineage‐specific metabolic delay should be considered when using unguided hPSC‐derived COs for downstream metabolite analyses.

hPSC‐derived CO NMR spectra had abundant hTau and lacked taurine, sharply contrasting with adult human and rodent brains, where taurine is abundant and hTau is absent. However, the increase in taurine with tissue maturation is a metabolic signature observed in the fetal brain [24, 25], supporting the use of COs as models for early brain development. Taurine biosynthesis involves a stepwise oxidation pathway that is not uniformly preserved in cell lines [31]. hTau serves as the immediate precursor, but its synthesis into taurine relies on oxidative mechanisms within developing astrocytes [31, 32]. The efficient oxidation of hTau to taurine marks a later developmental transition, which likely accounts for the accumulation of the precursor hTau and the absence of taurine in these stages.

A few studies have performed other variants of NMR spectroscopy in *human* organoids, particularly researching cancer. Knitch et al. [33] used an NMR‐based approach in cell spheroids. However, unlike the current study, Knitch et al. used thymic carcinoma spheroids, which were not neural. In agreement with our own finding of elevated lactate in COs, Knitch et al. revealed exceedingly high lactate and low glucose concentrations toward the oxygen‐depleted center of Ty82 cancer spheroids. More recently, Sapir et al. used ^13^C and ^31^P NMR spectroscopy to assess the metabolic rate of LDH in COs^35^. A study by Diserens et al. performed HR‐MAS NMR in 3D *rat* brain aggregates. Similar to our findings, they observed an abundance of lactate and hTau [34]. Finally, a study conducted by Van der Kemp et al. [35] used HR‐MAS NMR spectroscopy in colorectal cancer organoids. Although they found that metabolic measures are highly correlated between cancer organoids and tissue extracts, the study also highlights the benefits of studying intact tissue because it is non‐destructive and enables follow‐up analyses using omics approaches [10].

Our findings demonstrate that hPSC‐derived COs share metabolic similarities with human brain tissue, exhibiting features suggestive of a fetal developmental stage. The challenge of directly studying brain metabolism in human participants necessitates the use of model systems. With the use of animal models under increasing scrutiny, COs will likely be an important model system for brain metabolic studies in the future. Despite metabolic differences between COs and adult human brain, COs provide several important advantages as a human experimental model. They are suitable for histological and gene transcription assays, thereby enabling studies of the complex relationships between cellular organization, gene transcription, and metabolism. The feasibility of precise genetic modification in COs allows for the study of causal links between specific genes and their resulting brain metabolic profiles. Our results highlight that HR‐MAS NMR spectroscopy in COs is a viable approach for such metabolic studies.

## 5 Limitations and Future Work

Efforts were made to promote survival of the COs during the experiment. Namely, a relatively low spinning rate of 2500 Hz was chosen to minimize physical stress, and the sample temperature was maintained at 5°C to minimize metabolic degradation. However, oxygen and nutrient levels were not maintained during the NMR scan, and we did not assess the viability of the COs following the HR‐MAS experiment. Therefore, future work will involve (1) exploring methods to maintain oxygen and nutrient delivery during the scan, and (2) assessing cell viability following HR‐MAS to better understand the impact of the experiment on CO cell survival.

It is possible that scanning the organoids in pure D_2_O may have caused a cell swelling or lysis effect. Future work will aim to further optimize scanning protocols, including the scanning solvent, to better promote cell viability.

Despite collecting measurements at a range of CO age/maturity stages (85–312 days), we did not assess metabolic changes associated with organoid age/maturation. Rather, the scope of this study was limited to describing the methodology and demonstrating the feasibility of HR‐MAS NMR spectroscopy in hPSC‐derived COs. Future assessment of age‐related metabolic changes in COs is planned and will require a larger sample size, ideally with multiple replicates per organoid age.

## 6 Conclusion

HR‐MAS NMR yields detailed spectra, enabling quantitative estimation of metabolite levels in intact hPSC‐derived COs. Our findings suggest that COs exhibit some metabolic similarities to *in vivo* fetal human brain, with features such as high lactate, approximately equimolar Glu and Gln levels, low NAA, and the presence of hTau (and absence of Tau). This study demonstrates that HR‐MAS NMR spectroscopy is a useful methodology for assessing neurochemistry and metabolism in hPSC‐derived COs. As new CO protocols and disease models emerge, HR‐MAS NMR may help to identify disease‐modifying metabolic differences. Future “omic” analyses may further reveal how specific cellular, genetic, and gene transcriptional alterations impact the observed metabolite levels. Finally, the application of this HR‐MAS NMR approach will enable studies to improve our understanding of the cell biology and physiology of human‐derived COs, thereby providing an opportunity to improve the engineering of COs to better model human brain physiology.

## Author Contributions

Conception and design of the study: M.A.C.B., J.N.; Pulse sequence design: A.S.; Generation of organoids: V.C.; Acquisition of data: R.G.B., R.S., and F.S.; Analysis and interpretation of results: M.A.C.B., C.B., M.W., C.S., and J.N.; Drafting of the manuscript: M.A.C.B. and J.N.; Approval of the submitted manuscript, and acceptance of responsibility for all aspects of the study in ensuring that questions related to the accuracy or integrity of any part of the work are appropriately investigated and resolved: M.A.C.B., V.C., R.G.B., C.B., M.W., R.S., F.S., A.S., C.S., and J.N.

## Funding

This work is supported by the Canadian Institutes of Health Research (J.N., A.S., and C.S., Grant #: PJT‐183715) and the New Frontiers in Research Fund (NFRFT‐2022‐00327).

## Conflicts of Interest

The authors declare no conflicts of interest.

## Supporting information


**Figure S1:** Cerebral organoids at early stage of maturation. At Days 18 and 30, COs had neural rosette‐like structures, with an inner ventricular zone labeled by SOX2, a neural progenitor cell marker. TUJ1 + immature neurons were dispersed throughout the CO‐rosettes, but progressively migrated outward, forming an outer layer above the ventricular zone.
**Figure S2:** Metabolite concentrations in 85‐ to 312‐day‐old hPSC‐derived COs. Concentration of metabolites over time was recorded in 12 intact hPSC‐derived COs scanned between 85 and 312 days old. A barplot was generated with average concentrations of all detected metabolites. Barplot data represent the means ± SEM for 12 independent experiments. Ala: alanine; Pt: putrescine; Ace + GABA: acetate and GABA; NAA: *N*‐acetylaspartate; Leu: leucine; Val: valine; etOH: ethanol; Lac: lactate; Cho: choline; PCh + GPC: phosphocholine and glycerophosphocholine; Gly: glycine; mIns: myo‐inositol; Glu: glutamate; Gln: glutamine; hTau: hypotaurine; Cr: creatine; and β‐Glc: glucose.
**Table S1:** Parameters of the HR‐MAS NMR study in hPSC‐derived COs (minimum reporting standards in MRS^8^).
**Table S2:** Quantification of metabolites in 12 hPSC‐derived COs at different stages of maturation.
**Table S3:** Metabolite concentrations in hPSC‐derived COs compared with reference values from the human adult and fetal brain.

## Data Availability

The data that supports the findings of this study are available in the Supporting Information of this article. The raw data and processed diffusion edited spectra are available from the corresponding author upon request.
