# Supplementary material for: Metabolic Assessment in Human Pluripotent Stem Cell‐Derived Cerebral Organoids Using HR‐MAS NMR Spectroscopy
Source: NMR Biomed. 2026 Jun 26;39(8):e70343. doi: 10.1002/nbm.70343 (PMC13307635; doi:10.1002/nbm.70343)
Supplement: Supplementary file 1 — Figure S1: Cerebral organoids at early stage of maturation. At Days 18 and 30, COs had neural rosette‐like structures, with an inner ventricular zone labeled by SOX2, a neural progenitor cell marker. TUJ1 + immature neurons were dispersed throughout the CO‐rosettes, but progressively migrated outward, forming an outer layer above the ventricular zone. Figure S2: Metabolite concentrations in 85‐ to 312‐day‐old hPSC‐derived COs. Concentration of metabolites over time was recorded in 12 intact hPSC‐derived COs scanned between 85 and 312 days old. A barplot was generated with average concentrations of all detected metabolites. Barplot data represent the means ± SEM for 12 independent experiments. Ala: alanine; Pt: putrescine; Ace + GABA: acetate and GABA; NAA: N‐acetylaspartate; Leu: leucine; Val: valine; etOH: ethanol; Lac: lactate; Cho: choline; PCh + GPC: phosphocholine and glycerophosphocholine; Gly: glycine; mIns: myo‐inositol; Glu: glutamate; Gln: glutamine; hTau: hypotaurine; Cr: creatine; and β‐Glc: glucose. Table S1: Parameters of the HR‐MAS NMR study in hPSC‐derived COs (minimum reporting standards in MRS8). Table S2: Quantification of metabolites in 12 hPSC‐derived COs at different stages of maturation. Table S3: Metabolite concentrations in hPSC‐derived COs compared with reference values from the human adult and fetal brain. [file NBM-39-e70343-s001.docx]

**Supplementary materials**

## Method details

## Cerebral organoid formation

We adapted our CO differentiation protocol from a previously published study^1^. Briefly, hESC colonies were dissociated with Gentle cell dissociation reagent (GCDR, Stem Cell Technologies, 07174) for 7 mins at 37 °C, and 12,000 cells in 100 µL STEMdiff kit EB formation media (Stem Cell Technologies, 08570) supplemented with 50 µM Rock inhibitor Y-27632 (Stem Cell Technologies, 72302) were plated in 96 well V-bottom plate (low-binding) (Greiner bio-one, 651970). This time point was called day 0 of embryoid body (EB) formation. On day 1 and 3 of EB formation, 2 µM Dorsomorphin (Stem Cell Technologies, 72102) and 2 µM A83-01 (Stem Cell Technologies, 72022) to the EB formation media. On day 5, single EBs were transfer to individual wells of 24-well ultra-low attachment plate (Corning, 3473), and media was replaced with STEMdiff kit Induction media containing 1 µM SB431542 (Stem Cell Technologies, 72234) and 1 µM CHIR99021 (Stem Cell Technologies, cat. no. 72054) and cultured for 4 more days. On day 9, COs were placed onto a single dimple of an embedding sheet (Stem Cell Technologies, cat. no. 08579) and 15 µL of Matrigel (Corning, cat. 354277) was added to each organoid to encapsulate it. Matrigel droplet-containing COs were incubated at 37 °C for 30 min before 10-12 COs were washed into one well of a 6-well ultra-low attachment plate (Stem Cell Technologies, 38071) containing STEMdiff kit Expansion media containing 1 µM SB431542 and 1 µM CHIR99021. On day 13, individual organoids were transferred to each well of a 12-well miniature spinning bioreactor^1^ (3Dnamics #3DNA01, Customized spin omega bioreactor-12 well) containing STEMdiff kit Maturation media. From day 30 to day 60, extracellular matrix proteins were supplemented in Maturation media by dissolving Matrigel at 1% (v/v) containing human recombinant brain-derived neurotrophic factor (BDNF; PeproTech, AF-450-02). From day 61 onwards, COs were maintained by replacing Maturation media twice a week until the experimental collection time points, as indicated.

## Human embryonic stem cells maintenance

The use of human embryonic stem cells (hESCs) was approved by the Canadian Institutes of Health Research (CIHR) Stem Cell Oversight Committee (SCOC) and the Sunnybrook Research Ethics Board (REB - PIN: 1884). hESCs (H1/WA01) were purchased from WiCell Research Institute, Wisconsin, USA. ESCs were maintained in 5% CO2 incubators at 37°C. hESCs were cultured under feeder-free conditions in mTeSR Plus media (Stem Cell Technologies, 100-0276) on plates coated with Matrigel (Corning, 354277). Versene (Thermo Fisher Scientific, 15040-066) was used to dissociate hESCs into smaller clumps by manual pipetting every 4-5 days for maintenance. hESCs were routinely tested for quality control with a qPCR-based human stem cell pluripotency detection kit (Sciencell, 0853) and hPSC genetic analysis kit (Stem Cell Technologies, 07550).

## Cerebral organoid processing and immunostaining

COs were rinsed with ice-cold phosphate-buffered saline (PBS, without Ca^2+^ and Mg^2+^) (Wisent, 311-010-CL), fixed in 4% paraformaldehyde (PFA, Electron Microscopy Sciences, 19208) in PBS at 4ºC overnight. PFA was rinsed off using three washes for 5 min in PBS before cryopreservation by immersion in 20% sucrose (Sigma, 84097)/1X PBS at 4ºC overnight. COs were embedded in optical cutting temperature (O.C.T™) compound (Tissue-Tek®, Sakura Finetek U.S.A. Inc., Torrance, CA) on dry ice and stored at -80ºC. 10-micron sections were collected with a Leica CM3050 cryostat (Leica Microsystems Canada Inc., Richmond Hill, ON, Canada). Samples were collected on Fisherbrand^TM^ Superfrost^TM^ Plus Microscope Slides (Thermo Fisher Scientific, 12-550-15). Cryosections of fixed COs were washed in 0.1% Triton X-100 (Sigma, T8787) in PBS (PBST), then blocked for 1 hour at room temperature in 10% horse serum (HS, Wisent, 065-150) in PBST. Primary antibodies were diluted in blocking solution as follows: TUJ1 (1:500, BioLegend #802001), GABA (1:500, Sigma #A2052), GFAP (1:500, Novus #100-53809) and VGLUT1/SCL17A7 (1:2000, Synaptic Systems #135 302). After one hour at room temperature, slides were washed 5 times for 5 min in PBST and incubated with 1:500 dilutions of species-specific secondary antibodies (Invitrogen Molecular Probes) for 1 hour at room temperature. Slides were washed five times in PBST and counterstained with 4’,6-diamidino-2-phenylindole (DAPI, Invitrogen, D1306) and mounted in Aqua-polymount (Polysciences Inc., 18606-20). All images were taken using a Leica DMi8 Inverted Microscope (Leica Microsystems CMS, 11889113).


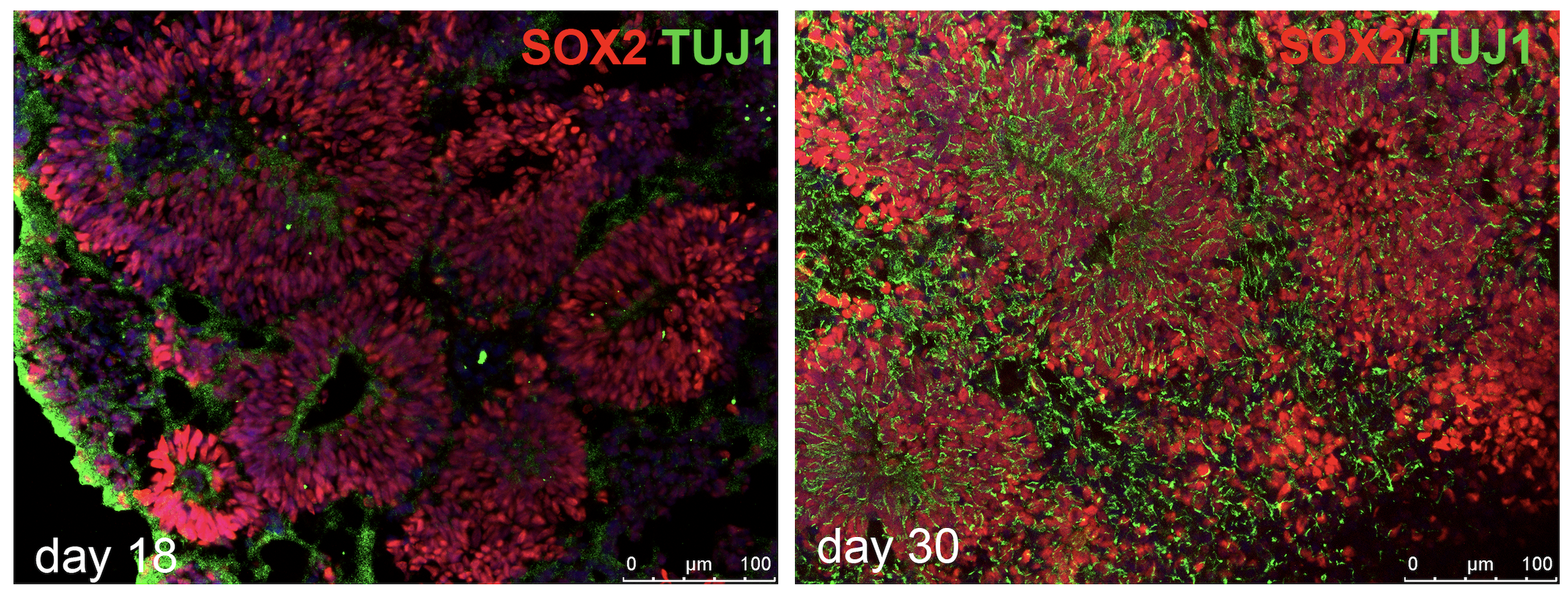


**Supplementary Figure 1. Cerebral organoids at early stage of maturation.** At day 18 and day 30, COs had neural rosette-like structures, with an inner ventricular zone labeled by SOX2, a neural progenitor cell marker. TUJ1+ immature neurons were dispersed throughout the CO-rosettes, but progressively migrated outward, forming an outer layer above the ventricular zone.

## Organoid sample preparation for HR-MAS NMR spectroscopy

For each NMR data acquisition, an individual intact CO was transferred directly from culture to the NMR rotor post-harvest without freezing. Intact COs were used for NMR analysis. 1H HR-MAS was performed using a 50 μL reduced-volume rotor (4 mm; HZ07213, Bruker, Billerica, MA, USA). After placing the organoid in the rotor, the remainder of the rotor volume was filled with D_2_O to provide a deuterium frequency lock.

Metabolite quantification was normalized to the individual volume of each organoid. The wet weight of each organoid was determined prior to the NMR scan using an analytical balance, and the volume was subsequently estimated using an assumed tissue density of 1 g/mL, consistent to reported brain tissue densities^2^. Organoid weights ranged from 3.9 mg (85 days old) to 15.8 mg (312 days old).

## Extended discussion

## *Cellular identity in COs*

## Although we did not verify the cellular identity of CO tissues in the current study, our group has previously assessed cellular identity in organoids generated using an identical protocol. Specifically, neural identity and function were validated by IHC detection of GABA β-subunits and VGLUT1 receptors, confirming the presence of functional neurons within hPSC-derived COs^3^.

Further insights into cell type distributions in COs can be gained from single-cell RNA sequencing studies in literature. Specifically, Sivitilli et al^4^, who generated organoids using protocols very closely related to our own, showed that at earlier organoid developmental stages (12-18 weeks), these organoids are composed predominantly of neuronal populations (~45-55%), together with substantial fractions of progenitor cells (~30-35%), and a smaller glial linage component (~10-15%). At these time points, neuronal populations appear to be largely excitatory in identity, as expression of canonical interneuron markers (e.g., DLX1, GAD1/2) is mostly absent. This overall composition (i.e., a large neuronal population, a substantial progenitor pool and a relatively smaller glial fraction) is broadly consistent with early-to-mid stages of corticogenesis, during which neurogenesis predominates and gliogenesis is only beginning to emerge^5^.

At later organoid developmental stages (~24 weeks), the emergence of interneuron-lineage transcriptional signatures is observed, with approximately 20% of cells expressing the canonical interneuronal marker DLX1^4^. This temporal pattern, with GABAergic neuronal populations emerging in later stages of organoid maturation, is also consistent with human cortical development wherein excitatory neurogenesis precedes the integration of inhibitory neurons^6,7^.

It should be noted that numerous organoid generation protocols exist, and these findings should be viewed as specific to unguided Lancaster protocol used here. Future work should aim to assess how divergent cell lineages and culturing methodologies modulate the metabolic and neurochemical profile.

*Presence of Ethanol in HR-MAS NMR spectra from COs*

In addition to main spectral differences, additional differences exist between COs and human brain spectra. Ethanol is rarely detected *in vivo* in the human brain due to its low physiological concentration. In contrast, CO spectra show a prominent triplet at 1.18 ppm, consistent with ethanol. The presence of ethanol in COs may arise from exogenous sources, such as 2-mercaptoethanol, a component of the organoid generation kit. Further validation is needed to clarify the source and persistence of the ethanol signal in hPSC-derived CO NMR spectra.

*Scope of research and future work*

While the current study focuses on the validation of a 1H HR-MAS methodology in COs, this approach provides a useful framework for longitudinal analyses of maturation-dependent metabolic changes in human brain models. The robust detection of metabolites demonstrated in COs suggests that HR-MAS is a viable tool for investigating both CO development and possible metabolic alterations in human disease models.

###
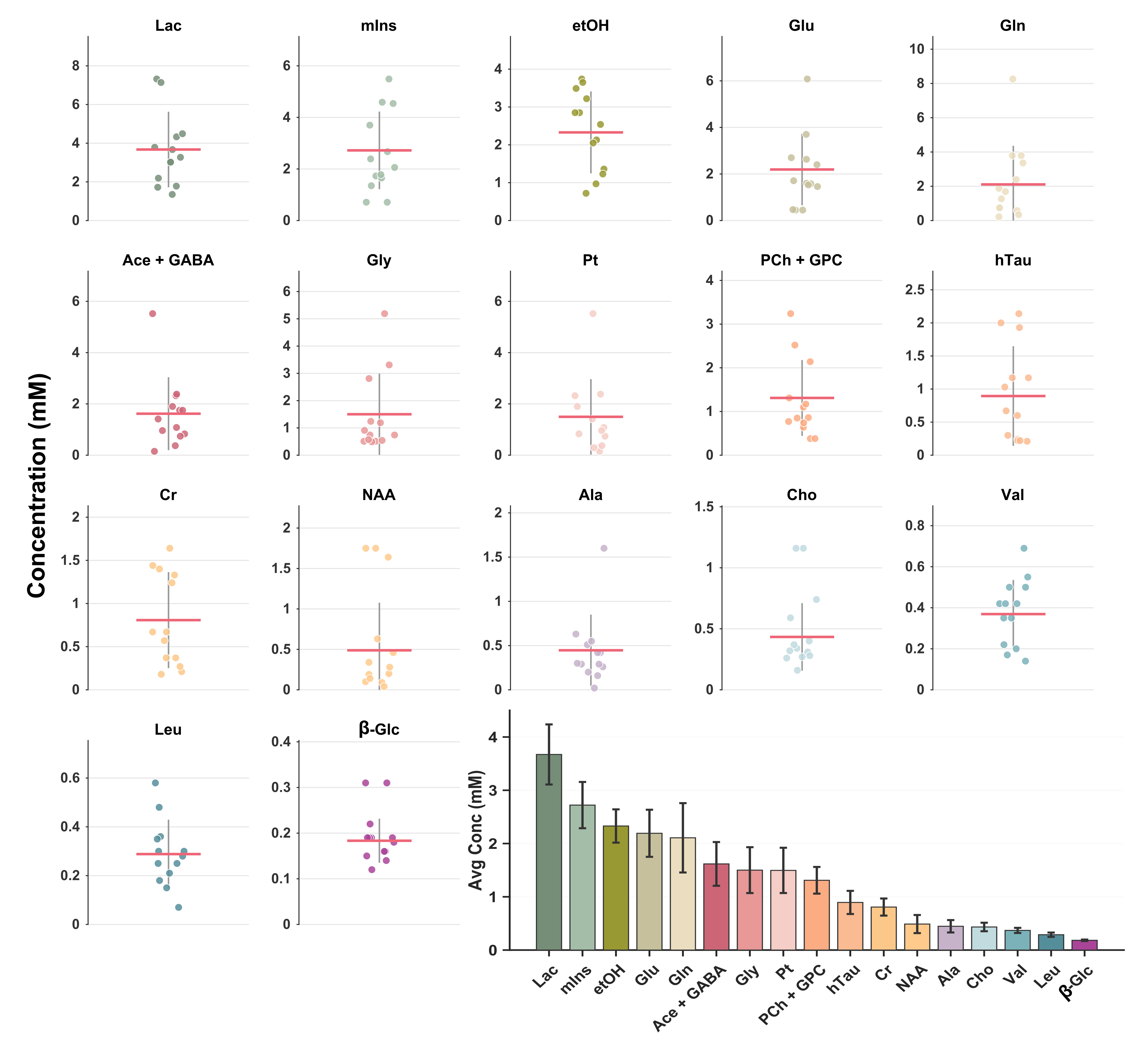


### Supplementary Figure 2. Metabolite concentrations in 85–312-day-old hPSC-derived COs. Concentration of metabolites over time was recorded in 12 intact hPSC-derived COs scanned between 85 and 312 days old. A barplot was generated with average concentrations of all detected metabolites. Barplot data represent the means ± SEM for 12 independent experiments. Ala: alanine; Pt: putrescine; Ace + GABA: acetate and GABA; NAA: N-acetyl-aspartate; Leu: leucine; Val: valine; etOH: ethanol; Lac: lactate; Cho: choline; PCh+GPC: phosphocholine and glycerophosphocholine; Gly: glycine; mIns: myo-inositol; Glu: glutamate; Gln: glutamine; hTau: hypotaurine; Cr: creatine; and β-Glc: glucose.

**Supplementary Table 1. Parameters of the HR-MAS NMR study in hPSC-derived COs (Minimum Reporting Standards in MRS**^8^**).**

| Site: Department of Chemistry, University of Toronto Scarborough | |
| --- | --- |
| 1. Hardware |  |
| a. Field strength [T] | 500 MHz (11.7 T) |
| b. Manufacturer | Bruker (Billerica MA, USA) |
| c. Model (software version if available) | Avance III 500 MHz ^1^H spectrometer |
| d. RF coils: nuclei (transmit/ receive), number of channels, type, body part | 4 mm Comprehensive Multiphase probe (Bruker Switzerland AG, Fällanden, Switzerland) and magic-angle gradient. |
| e. Additional hardware | - 2500 Hz Spinning rate  - 50 µl reduced volume Teflon rotor with D2O for locking.  - Temperature maintained at 5^o^C |
| 2. Acquisition |  |
| a. Pulse sequence | Diffusion editing experiments used a bipolar pulse pair longitudinal eddy-current delay (BPP-LED) sequence^9^ with a Gradient pulse of 2.4ms at ~60 Gauss/cm and a diffusion time of 120 ms. |
| b. Volume of Interest (VOI) locations | N/A |
| c. Nominal VOI size [cm^3^, mm^3^] | N/A |
| d. Repetition Time (TR), and diffusion parameters. | -TR of 11 s (5 x T1 of longest relaxing peak) to eliminate T1 weighting allowing for absolute quantification  -Diffusion gradient width = 2.4ms  -Diffusion pulse pair separation 𝜏 = 300 μs  -Diffusion time Δ= 120 ms  -Eddy Current Delay= 5ms |
| e. Total number of Excitations or acquisitions per spectrum | 512 total scans |
| f. Additional sequence parameters  (Spectral width in Hz, number of spectral points, frequency offsets)  If MRSI: 2D or 3D, FOV in all directions, matrix size, acceleration factors, sampling method | - 10,000 Hz spectral width  - 16,484 time-domain points  - 8 dummy scans |
| g. Water Suppression Method | Water pre-saturation was prepended to all 1D experiments with an effective B1 field of 100Hz |
| h. Shimming Method, reference peak, and thresholds for “acceptance of shim” chosen | Automated B0 field mapping followed by manual shimming |
| 3. Data analysis methods and outputs |  |
| a. Analysis software | TopSpin 4.3 (Bruker). |
| b. Processing steps deviating from quoted reference or product | None. |
| c. Output measure | Absolute concentration using external reference. |
| d. Quantification references and assumptions, fitting model assumptions | Peak integration was performed using TopSpin 4.3 (Bruker). A 10 mM external alanine standard was used to convert signal intensity to concentration. Quantification was achieved according to Equation 1, which corrects for the number of protons contributing to each NMR peak, and results were adjusted for each CO’s volume, as described in the methods section: *HR-MAS NMR metabolite* *assignment and quantification.* |
| 4. Data Quality |  |
| a. Reported variables  (SNR, Linewidth with reference peaks) | SNR = 192.775 ± 153.346;  Linewidth max linewidth = 3.650 Hz, mean = 2.6813 ± 0.5806 Hz  SNR was calculated using the amplitude of the largest peak of the spectra over the standard deviation of the noise.  Linewidth was calculated using the mean peak width of Cr at 3.014 ppm |
| b. Data exclusion criteria | *No organoids excluded* |
| c. Quality measures of postprocessing Model fitting (e.g. CRLB, goodness of fit, SD of residual) | *N/A* |
| d. Sample Spectrum | Figure 3. ^1^H HR-MAS NMR Spectroscopy in hPSC–derived COs |

**Supplementary Table 2**. **Quantification of metabolites in 12 hPSC-derived COs at different stages of maturation.**

**
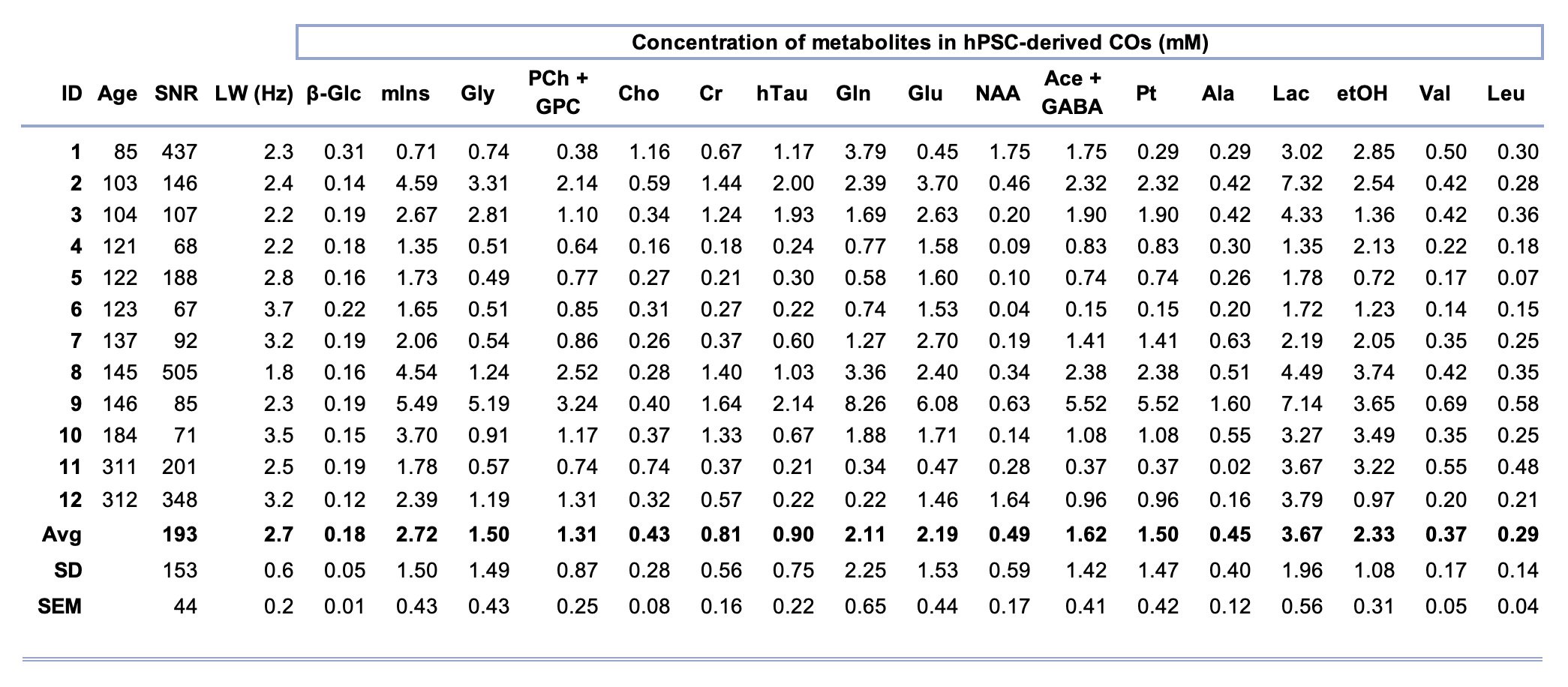
**

**Supplementary Table 3** **Metabolite concentrations in hPSC-derived COs compared to reference values from the human adult**^10,11^ **and fetal brain**^12–14^**.**

**
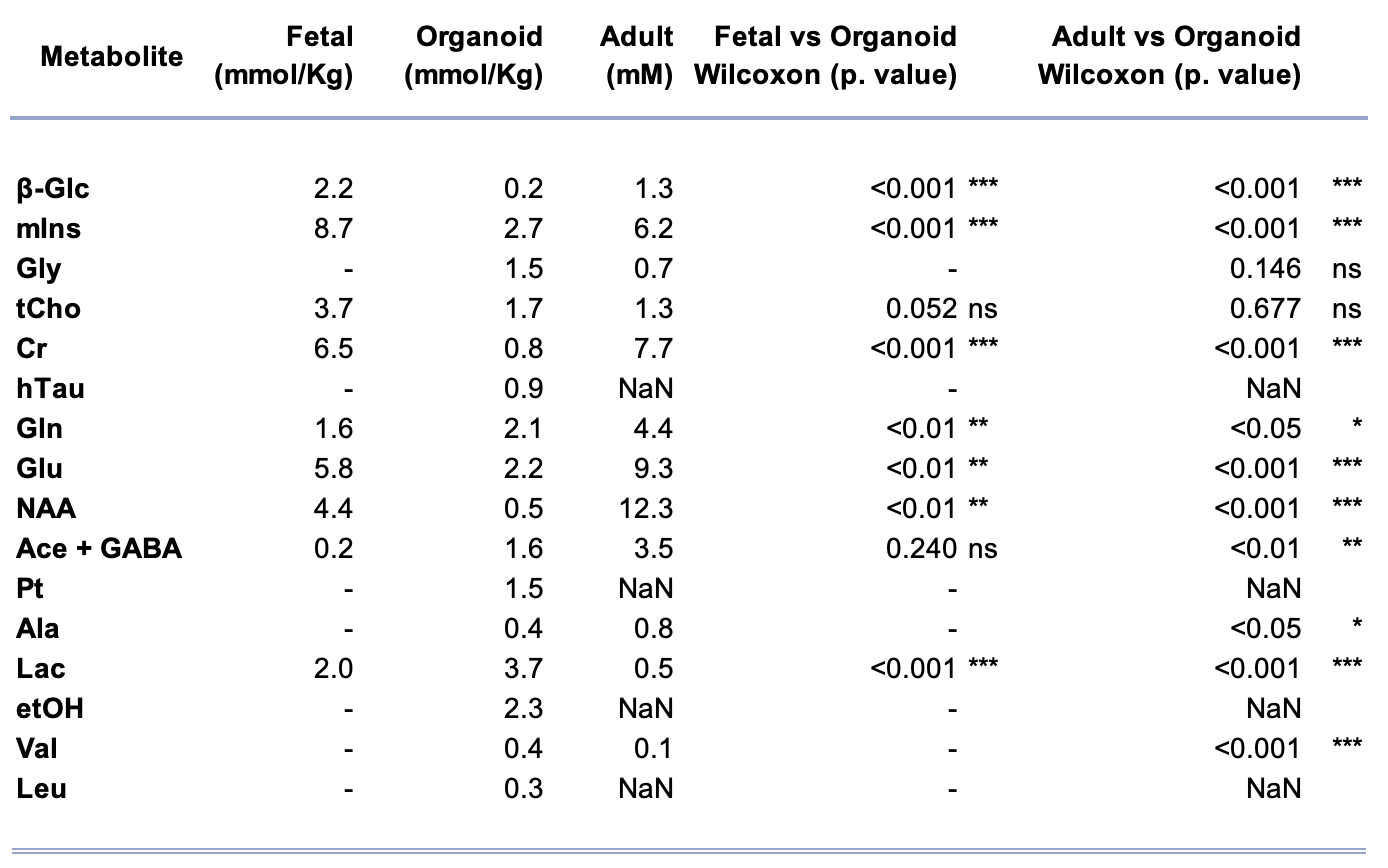
**

**Supplemental references**

1. Qian X, Jacob F, Song MM, Nguyen HN, Song H, Ming GL. Generation of human brain region-specific organoids using a miniaturized spinning bioreactor. *Nat Protoc*. 2018;13(3):565-580. doi:10.1038/nprot.2017.152

2. DiResta GR, Lee J, Arbit E. Measurement of brain tissue specific gravity using pycnometry. *Journal of Neuroscience Methods*. 1991;39(3):245-251. doi:10.1016/0165-0270(91)90103-7

3. Vander Heiden MG, Cantley LC, Thompson CB. Understanding the Warburg Effect: The Metabolic Requirements of Cell Proliferation. *Science*. 2009;324(5930):1029-1033. doi:10.1126/science.1160809

4. Sivitilli AA, Gosio JT, Ghoshal B, et al. Robust production of uniform human cerebral organoids from pluripotent stem cells. *Life Sci Alliance*. 2020;3(5):e202000707. doi:10.26508/lsa.202000707

5. Kriegstein A, Alvarez-Buylla A. The glial nature of embryonic and adult neural stem cells. *Annu Rev Neurosci*. 2009;32:149-184. doi:10.1146/annurev.neuro.051508.135600

6. Marín O. Interneuron dysfunction in psychiatric disorders. *Nat Rev Neurosci*. 2012;13(2):107-120. doi:10.1038/nrn3155

7. Fishell G, Kriegstein AR. Neurons from radial glia: the consequences of asymmetric inheritance. *Curr Opin Neurobiol*. 2003;13(1):34-41. doi:10.1016/s0959-4388(03)00013-8

8. Lin A, Andronesi O, Bogner W, et al. Minimum Reporting Standards for in vivo Magnetic Resonance Spectroscopy (MRSinMRS): Experts’ consensus recommendations. *NMR in Biomedicine*. 2021;34(5):e4484. doi:10.1002/nbm.4484

9. Wu DH, Chen AD, Johnson CS. An Improved Diffusion-Ordered Spectroscopy Experiment Incorporating Bipolar-Gradient Pulses. *Journal of Magnetic Resonance, Series A*. 1995;115(2):260-264. doi:10.1006/jmra.1995.1176

10. de Graaf R. *In Vivo NMR Spectroscopy*. Vol 2. 1st ed. John Wiley & Sons, Ltd; 2019. doi:10.1002/9781119382461

11. Govindaraju V, Young K, Maudsley AA. Proton NMR chemical shifts and coupling constants for brain metabolites. *NMR Biomed*. 2000;13(3):129-153. doi:10.1002/1099-1492(200005)13:3%3C129::aid-nbm619%3E3.0.co;2-v

12. Tomiyasu M, Aida N, Endo M, et al. Neonatal Brain Metabolite Concentrations: An In Vivo Magnetic Resonance Spectroscopy Study with a Clinical MR System at 3 Tesla. *PLOS ONE*. 2013;8(11):e82746. doi:10.1371/journal.pone.0082746

13. Hüppi PS, Posse S, Lazeyras F, Burri R, Bossi E, Herschkowitz N. Magnetic resonance in preterm and term newborns: 1H-spectroscopy in developing human brain. *Pediatr Res*. 1991;30(6):574-578. doi:10.1203/00006450-199112000-00017

14. Kreis R, Hofmann L, Kuhlmann B, Boesch C, Bossi E, Hüppi P s. Brain metabolite composition during early human brain development as measured by quantitative in vivo 1H magnetic resonance spectroscopy. *Magnetic Resonance in Medicine*. 2002;48(6):949-958. doi:10.1002/mrm.10304
